# Serum Pro-N-Cadherin Correlates With Cardiac Injury in the Radiation Late Effects Cohort of Nonhuman Primates

**DOI:** 10.1016/j.jacadv.2026.103050

**Published:** 2026-07-23

**Authors:** Paul D. Ferrell, Drew Neish, Gregory O. Dugan, George W. Schaaf, John D. Olson, Kristianne M. Oristian, Kristofer T. Michalson, Donna Niedzwiecki, Dalane Kitzman, Thomas C. Register, J. Mark Cline, Salvatore V. Pizzo, Chang-Lung Lee

**Affiliations:** a Department of Pathology, Duke University School of Medicine, Durham, North Carolina, USA; b Department of Radiation Oncology, Duke University School of Medicine, Durham, North Carolina, USA; c Biostatistics Shared Resource, Duke Cancer Institute, Durham, North Carolina, USA; d Department of Pathology, Section on Comparative Medicine, Wake Forest University School of Medicine, Winston-Salem, North Carolina, USA; e Department of Biostatistics and Bioinformatics, Duke University, Durham, North Carolina, USA; f Department of Internal Medicine, Sections of Cardiovascular Medicine and Gerontology and Geriatric Medicine, Wake Forest University School of Medicine, Winston-Salem, North Carolina, USA

**Keywords:** cardiac fibrosis, diastolic dysfunction, pro-N-cadherin, radiation-related heart disease, serum biomarker

## Abstract

**Background:**

The delayed effects of radiation exposure on the heart often manifest as cardiac fibrosis and diastolic dysfunction, which can develop years after exposure. However, no Food and Drug Administration-approved serological biomarker is available to assess an individual’s risk of developing radiation-related heart disease (RRHD).

**Objectives:**

The authors hypothesize that the risk of RRHD can be assessed using serum pro-N-cadherin (PNC), a promising marker for predicting subclinical heart failure in the general population.

**Methods:**

We examined 46 male nonhuman primates (NHPs) that survived total-body irradiation and 10 unirradiated controls. NHPs exhibited cardiac fibrosis scores ranging from less severe (F0-1) to more severe (F2-3). Cardiac tissue samples collected at necropsy, with a median of 6.8 years post-irradiation, were stained for PNC by immunohistochemistry. PNC was quantified in longitudinal serum samples collected 2, 1, and 0 years before necropsy. The associations of serum PNC levels with cardiac fibrosis scores and echocardiographic parameters were examined.

**Results:**

Histological examinations showed aberrant localization of PNC in NHPs with cardiac fibrosis. Elevated serum PNC levels were associated with severe cardiac fibrosis (F2-3) (area under the curve = 0.81, *P* = 0.006) and echocardiogram parameters of diastolic dysfunction, including lateral e’ and lateral E/e’ (*P* < 0.05). Cardiac fibrosis was associated with the greatest elevation in serum PNC among measured comorbidities.

**Conclusions:**

Our findings from this radiation late effects cohort of NHPs reveal a strong association between elevated serum PNC and cardiac injury. These findings pave the way for future clinical studies to develop serum PNC as a biomarker of RRHD in humans.

Radiation-related heart disease (RRHD) is a serious and often under-recognized complication of radiation therapy (RT), particularly in patients treated for cancers located near the chest, such as breast, lung, or mediastinal cancers.^1^ For example, while adjuvant RT improves overall survival in patients with invasive breast cancer, survivors who receive RT are at increased risk of late cardiac morbidity and mortality.^2^, ^3^, ^4^, ^5^ Risk-guided cardioprotection treatments of cancer patients receiving cardiotoxic therapies have shown promise in early-stage clinical trials.^6^ Numerous papers reveal a positive association between the risk of heart disease and total radiation doses to the heart and different cardiac substructures.^7^, ^8^, ^9^, ^10^ Besides cancer patients who receive thoracic RT, epidemiologic studies from atomic bomb survivors also reveal a significant dose-related increase in the risk of cardiovascular disease.^11^ For example, one multicenter study reported an estimated excess relative risk per Gy was 14% for heart disease.^12^ While much of the focus in radiation-induced cardiovascular injury has historically been on left ventricular (LV) systolic dysfunction, recent research has highlighted the prominence of diastolic dysfunction as a key manifestation of RRHD.^13^, ^14^, ^15^, ^16^, ^17^ Diastolic dysfunction refers to the impairment in the heart's ability to relax and fill properly during the diastolic phase, leading to increased filling pressures and reduced cardiac efficiency, even in the absence of overt systolic dysfunction.

Given that radiation-induced diastolic dysfunction may occur in the absence of noticeable systolic impairment, it presents a unique challenge in diagnosis and management. The pathophysiology of RRHD is multifactorial.^18^ Radiation exposure to the heart induces inflammation, vascular injury, and fibrosis, all of which contribute to the stiffening of the left ventricle (LV) and impair its ability to relax during diastole.^17^ These changes result in altered LV compliance, elevated diastolic pressures, and eventually, the clinical symptoms of heart failure (HF), including shortness of breath, fatigue, and exercise intolerance. Traditional cardiac biomarkers, such as B-type natriuretic peptide (BNP) and troponin, are commonly used to assess HF and monitor cardiac function in a variety of conditions.^19^ However, natriuretic peptides and troponin levels measured early after RT have not consistently demonstrated effectiveness in detecting subclinical cardiotoxicity or predicting future cardiomyopathy.^1^^,^^20^^,^^21^ As a result, traditional biomarkers like BNP may not adequately reflect the nuanced and progressive nature of RRHD, especially in cases where diastolic dysfunction is the predominant feature. This limitation underscores the need for more sensitive and specific biomarkers tailored to detect early changes in cardiac function related to RT, as this condition can progress to more severe HF if left unaddressed.

Pro-N-cadherin (PNC) has emerged as a promising biomarker for predicting the early onset of HF and as an indicator of cardiac fibrosis.^22^, ^23^, ^24^ N-cadherin, a cell adhesion molecule, plays a crucial role in maintaining cardiac tissue integrity and function.^25^^,^^26^ Under pathological conditions, the defective processing of N-cadherin results in the aberrant localization of the precursor form to cardiac intercalated discs and its release into circulation, which in turn correlates with the extent of fibrosis in the myocardium.^22^, ^23^, ^24^ Thus, elevated levels of PNC reflect ongoing myocardial injury, fibrosis, and remodeling, which are early precursors to HF. Our previous publications indicate that measuring serum PNC levels could help identify patients at risk for developing HF, even before clinical symptoms appear, and may provide a more sensitive marker for cardiac fibrosis compared to traditional biomarkers.^22^^,^^23^ Common covariates such as age, body mass index (BMI), and sex were observed to have no significant impact on serum PNC. The goal of this study was to determine the potential of serum PNC for the early detection and monitoring of RRHD in the radiation late effects cohort of nonhuman primates (NHPs), which had been previously demonstrated to be at risk for RRHD and diastolic dysfunction.^27^, ^28^, ^29^, ^30^

## Materials and methods

### The Wake Forest NHP radiation late effects cohort

The NHPs involved in this study were selected from a larger group of Rhesus macaques (*Macaca mulatta*) as part of the Wake Forest University (WFU) Nonhuman Primate Radiation Late Effects Cohort (NHP RLEC).^29^, ^30^, ^31^, ^32^ This cohort has been built over the past 2 decades to study the long-term effects of radiation in a large animal model closely related to humans, sharing approximately 93% of their DNA. All NHPs used for this study were male due to the availability of longitudinally collected serum samples. The RLEC consists of about 200 living animals (both male and female) that are monitored daily and undergo clinical exams, routine blood and fluid collections, and noninvasive imaging such as magnetic resonance imaging, computed tomography (CT), dual-energy X-ray absorptiometry, and ultrasound. The cohort also includes around 130 deceased animals, which were euthanized upon reaching humane endpoints and subsequently underwent full necropsy examinations with the collection of both fixed and frozen tissues, as well as viable cells.

Of the animals in the RLEC, approximately 290 were exposed to radiation in various studies at different institutions to investigate radiation effects and medical countermeasures. Around 40 animals were never irradiated (IR) and served as controls. The control and IR animals were sourced from several institutions, including WFU, the University of Maryland, the University of Illinois, the Armed Forces Radiobiology Research Institute, the Lovelace Respiratory Research Institute, Citox Labs, Charles River Laval, and Primate Products. The IR animals in this study received total-body irradiation (TBI) doses ranging from 6 to 8.5 Gy under the oversight of the Institutional Animal Care and Use Committee at their respective institutions. Two irradiation strategies were employed: 1) linear accelerator-derived photons at a nominal mean energy of 2 MeV, delivered at 80 cGy/min in a split dose, with half delivered anterior-posterior and half posterior-anterior; or 2) cobalt 60-derived gamma irradiation, delivered bilaterally at 60 cGy/min simultaneously. These doses are potentially lethal: for Rhesus macaques, the LD_10/30_ is approximately 5.5 Gy, the LD_50/30_ is around 6.7 Gy, and the LD_90/30_ is approximately 8 Gy.^33^ Surviving animals were subsequently transferred to WFU for long-term postradiation monitoring. Previous reports have detailed the irradiation methods, supportive care strategies, and acute effects for many of the animals in this cohort.^33^, ^34^, ^35^ To model North American humans, the RLEC are fed a commercially available diet (5L0P, LabDiet) formulated to model the “typical American Diet.”

## Ethical approval

All in vivo blood collections and other postirradiation procedures were conducted at WFU with approval by the Institutional Animal Care and Use Committee of WFU. WFU has an Assurance on file in the Office for Protection from Research Risks, Office of the Director, National Institutes of Health, that accepts responsibility for the humane care and use of animals (OPRR #A-3391-01). The Laboratory Animal Care Program of the WFU School of Medicine complies with the “Principles for Use of Animals” and the “Guide for the Care and Use of Laboratory Animals” (National Research Council. 2011. Guide for the Care and Use of Laboratory Animals: Eighth Edition. Washington, DC: The National Academies Press), all provisions of the Animal Welfare Act, and has been accredited by the Association for Assessment and Accreditation of Laboratory Animal Care, International (AAALAC) since April 8, 1966 (AAALAC File #8).

### Echocardiography

NHPs were sedated with 10 to 15 mg/kg ketamine and 0.05 to 0.15 mg/kg midazolam, and echocardiography was performed using a Logiq S8 ultrasound system (GE Healthcare) by trained NHP ultrasonographers following guidelines for image acquisition and analysis established by the American Society of Echocardiography and European Association of Cardiovascular Imaging^36^^,^^37^ combined with recommendations for rhesus macaque echocardiograms reported by Korcarz et al^38^ as previously described.^28^^,^^39^ Captured images and video recordings were analyzed using Image-Arena software (TomTec Imaging Systems GMBH). Parasternal short and long axis, and apical 2- (A2C) and 4-chamber (A4C) views of the heart were obtained to evaluate structure and systolic and diastolic function. Motion (M) mode views at the level of the LV papillary muscles were captured to assess LV internal diameters at end diastole and end systole and fractional shortening. The LV endocardium was traced at end diastole and end systole in A2C and A4C views to obtain end diastolic and systolic volumes by the method of disks. The left atrium (LA) endocardium was traced at end systole in A2C and A4C views to calculate biplane LA end-systolic volume (LA Vol BP). Pulsed wave Doppler images taken at the mitral valve inflow tract in the A4C view were used to measure early (E) wave and late (A) wave peak filling velocities, E-wave deceleration slope, E-wave deceleration time, and the E/A ratio. Tissue Doppler imaging of the lateral annulus of the mitral valve in the A4C view was used to assess early (e’) and late (a’) mitral annular descent velocities, derive the e’/a’ ratio, and complete the calculation for the E/e’ ratio, an index of LV filling pressure.^40^ The LV outflow tract diameter (LVOT diam) at the beginning of systole was measured using the parasternal long-axis view. Body surface area (BSA, m^2^) at the time of the echocardiogram was calculated as: BSA = body weight^2/3^ × 0.0969, utilizing the animal’s body weight (kg).^41^

### Pathology and immunohistochemistry

**Figure 1:**
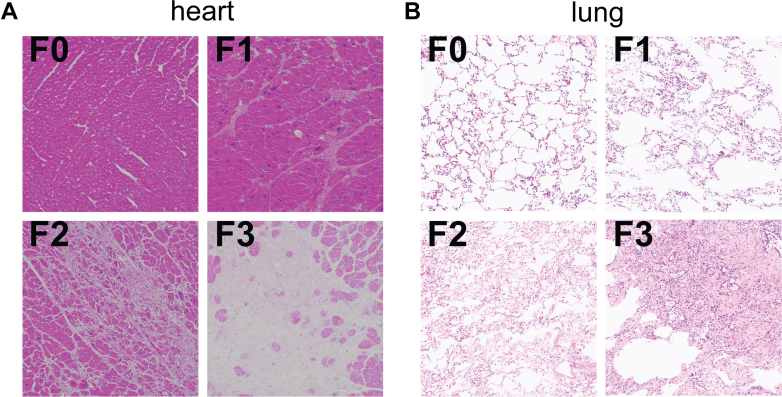
Representative Histology of the Fibrosis Scoring System (A) H&E-stained sections of heart from NHPs at 10x magnification. (F0) Normal myocardial tissue with no evidence of fibrosis. (F1) Minimal/mild myocardial fibrosis—scattered regions of the myocardium contain thin trabeculae of pale-pink-staining collagen that separate myocardial bundles. (F2) Moderate myocardial fibrosis—many regions of the myocardium contain variably thick streams of collagen that separate and replace cardiomyocytes. (F3) Marked/severe myocardial fibrosis—significant regions of the myocardium are effaced and replaced by dense accumulations of pale tan mature collagen. (B) H&E-stained sections of lung from NHPs at 10x magnification. (F0) Normal pulmonary tissue with no evidence of fibrosis. (F1) Minimal/mild pulmonary fibrosis—scattered regions of the pulmonary interstitium are expanded by thin trabeculae of pale-pink-staining collagen. (F2) Moderate pulmonary fibrosis—many regions of the pulmonary interstitium contain variably thick streams of pale-pink collagen. (F3) Marked/severe pulmonary fibrosis—significant regions of the pulmonary architecture are effaced and replaced by dense accumulations of pink, mature collagen. H&E = hematoxylin and eosin; NHP = nonhuman primate.

Animals were euthanized and taken to necropsy when they met predefined clinical criteria, such as a diagnosis of malignant neoplasia, or in rare cases, died unexpectedly. A complete necropsy was performed, followed by systematic histologic evaluation of all major organ systems and tissue types. Sections from the right and left ventricular free walls, as well as the intraventricular septum, were collected from the heart. Lung tissue was collected and photographed, and the right and left lungs were weighed individually. Six regions of the lung (left cranial/middle/caudal, right cranial/middle/caudal) were collected from each animal as directed by the supervising veterinary pathologist. If the lung lobe was normal in appearance, a section was chosen that included a grossly visible bronchus, artery, and vein. Heart and lung tissues were fixed in 4% neutral buffered formalin for 48 and 24 hours, respectively, and then transferred to 70% ethanol. Hematoxylin and eosin slides were routinely prepared and reviewed by board-certified veterinary pathologists. Tissue sections were classified as normal, mildly, moderately, or severely fibrotic.^42^ Cardiac and pulmonary fibrosis was qualitatively scored as none = F0, minimal/mild = F1, moderate = F2, and marked/severe = F3. See Figure 1 for representative histology of the scoring system. Immunohistochemistry (IHC) for staining PNC was performed following the protocol previously described.^23^

### Quantification of serum PNC by enzyme-linked immunosorbent assay

The detection and quantification of serum PNC by enzyme-linked immunosorbent assay (ELISA) were described previously.^23^ The recombinant pro domain of N-cadherin, amino acids 26 to 159 (Accession # AAB22854), was generated and supplied by GenScript and used to optimize a PNC sandwich ELISA. High-binding ELISA plates (Costar) were used to bind 1 μg/well of anti-PNC antibody 10A10 as the capture antibody. Washes were performed using phosphate-buffered saline (PBS) pH 7.4, 0.1% Tween 20. Three washes (300 μL/well) were performed between each of the following steps using a Biotek ELx405 Select CW automated plate washer. All steps were performed at room temperature with room-temperature equilibrated buffers. Capture antibody was bound overnight at room temperature in PBS pH 7.4, followed by blocking with 300 μL per well blocking buffer 5% nonfat dry milk (Bio-Rad) in 1× PBS (Gibco) with 0.1% Tween 20 for 1 hour. Serum samples and pro domain analyte standard were applied 100 μL per well in 1% BSA, PBS pH 7.4, 0.1% Tween 20 for 1 hour, followed by 100 μL/well 1:800 dilution of biotinylated polyclonal sheep α-PNC detection antibody (R&D BAF1388) in 1% BSA, PBS pH 7.4, 0.1% Tween 20 for 1 hour. Streptavidin horseradish peroxidase conjugate (Thermo Fisher) was applied at 100 μL per well, 1:800 in 2% BSA, PBS pH 7.4 for 20 minutes. Reagent 1-Step Turbo TMB-ELISA (Thermo Scientific, 34022) was substituted for detection following the supplier’s protocol. Plates were read using the Biotek Cytation 3 Imager Reader at an absorbance of 450 nm. Linear dilution of pooled Rhesus Macaque serum was used to verify the cross-reactivity of the assay (Supplemental Figure 1).

### Statistical analysis

Descriptive statistics were stratified by irradiation status and expressed as the median and IQR (Q1, Q3) for continuous variables, compared across strata with Mann-Whitney *U* tests, and frequency (N) and percentage (%) for categorical variables, compared across strata with Fisher exact tests.

The difference in PNC distributions between high cardiac fibrosis (F2-3) and low cardiac fibrosis (F0-1) was evaluated separately within IR and unirradiated NHPs at necropsy, as well as 1 and 2 years prior to necropsy. As cardiac fibrosis was assessed postmortem, fibrosis scores at earlier time points were inferred; to avoid biasing a repeated measures framework with this potential measurement error for time points further from necropsy, comparisons between high and low cardiac fibrosis groups were performed cross-sectionally at each time point using Mann-Whitney *U* tests. Samples with any missing longitudinal serum PNC measurements were excluded from this analysis. Receiver-operating characteristic analysis was performed within IR NHPs to determine the association between PNC and high cardiac fibrosis at necropsy.

Echocardiogram parameters were evaluated across 4 subcohorts: no IR, F0-1; no IR, F2-3; IR, F0-1; and IR, F2-3. The median (Q1, Q3) is presented for each measure stratified by subcohort, and measures were compared across all subcohorts using the one-way Kruskal-Wallis test, followed by Dunn’s post hoc tests with Bonferroni correction for multiple comparisons. Echocardiogram parameters did not consistently satisfy normality of residuals assumptions when examining differences across subcohort, precluding linear mixed model approaches; 2 cross-sectional analyses were therefore conducted: one including all observations within 30 months of necropsy and one restricted to each NHP’s single measurement closest to necropsy within 12 months to minimize the potential influence of multiple observations per subject. The latter analysis was restricted to IR NHPs due to insufficient sample size in the unirradiated group within this time window. As an exploratory complement, a proportional odds regression model was additionally fit for each echocardiogram parameter by subcohort,^43^^,^^44^ adjusting for BSA and age at necropsy, with the corresponding *P* value reported alongside the unadjusted result.

The relationship between PNC and a range of comorbidities, defined as the absence or presence of ever experiencing the comorbidity, was assessed using the Mann-Whitney *U* test, and the median (Q1, Q3) of the PNC distribution was stratified by the absence/presence of each comorbidity. The difference in median PNC between comorbidities was also determined. The comorbidities examined were: hypertension; overweight; underweight; tumor (carcinoma, sarcoma, or both); gastrointestinal; heart murmur; lung (defined by abnormal CT density); diabetes; cataracts; hepatic cysts; kidney (defined by cyst, nephromegaly, abnormal CT density, or abnormal blood urea nitrogen levels); arthritis; dermatitis; testicular atrophy; irradiation status; high pulmonary fibrosis; and high cardiac fibrosis. This analysis was limited to PNC at necropsy to ensure the reliability of the cardiac and pulmonary fibrosis scores. A proportional odds regression model was additionally fit for each comorbidity and PNC, adjusting for BSA and age at necropsy,^43^^,^^44^ with the corresponding *P* value reported alongside the unadjusted result. This analysis was additionally repeated within the IR subcohort and overall.

To examine the association between echocardiogram parameters and PNC, linear mixed models were fit with a random intercept for subject to account for repeated measures within each NHP. PNC was log-transformed prior to this analysis to address right skew and satisfy the distributional residual assumptions of the linear mixed model. Echocardiogram parameters were linked to the closest available PNC measurement for each NHP within a 6-month window. Each echocardiogram measure was a continuous fixed effect alongside time and an interaction term between the echo measure and time. Time was coded as 3 levels reflecting proximity to necropsy: t_0_ (<6 months), t_1_ (6-18 months), and t_2_ (>18 months). Models were fit separately for each echocardiogram parameter. Adjusted models included BSA and age at echocardiogram as covariates. For each model, the conditional effect of the echocardiogram parameter at t_0_ and the interaction with the time coefficient were determined, with 95% CIs and *P* values; the interaction term quantifies the association with log(PNC) as time progresses from necropsy. This analysis was reported within the IR subcohort and overall.

All statistical tests were 2-sided, with *P* < 0.05 considered statistically significant. Although these analyses were exploratory in nature, given the number of tests performed, a Bonferroni-adjusted significance threshold is additionally reported where relevant.

## Results

### Population dynamics of the NHP cohort

The cohort used in this study included 56 NHPs, 10 unirradiated (no IR) and 46 IR, and was selected based on irradiation status, cardiac fibrosis scoring, and sample availability (Table 1). Distribution of cardiac fibrosis scores among unirradiated and IR NHPs was subject to sample availability and request (Table 1). Subjects had a median age (Q1, Q3) of 11.7 (8.8, 16.4) at necropsy. Unirradiated NHPs were significantly older at necropsy than IR NHPs (no IR median age (Q1, Q3) = 17.4 (15.4, 19.7), IR median age (Q1, Q3) = 11.3 (8.7, 15.9), *P* = 0.044). Additionally, unirradiated NHPs had significantly higher calculated BSA relative to IR NHPs (no IR median BSA [m^2^] [Q1, Q3] = 0.52 [0.44, 0.60], IR median BSA [m^2^] = 0.36 [0.29, 0.44], *P* < 0.001). Among IR subjects, the median year since irradiation was 6.8 (5.5-11.0), and the median radiation dose was 6.7 (6.5-7.2) Gy. All IR NHPs received TBI.

**Table 1:** Demographic Summary of the NHP Cohort

	Overall (N = 56)	Unirradiated (n = 10)	Irradiated (n = 46)	*P* Value
Age at necropsy (y)				
Median (Q1, Q3)	11.68 (8.82, 16.44)	17.40 (15.42, 19.71)	11.30 (8.67, 15.85)	0.044
Missing	6	6	0	
BSA (m^2^)				
Median (Q1, Q3)	0.39 (0.30, 0.48)	0.52 (0.44, 0.60)	0.36 (0.29, 0.44)	<0.001
Missing	0	0	0	
IR dose (Gy)				
Median (Q1, Q3)	-	-	6.74 (6.50, 7.20)	-
Missing	-	-	0	
Time since irradiation (y)				
Median (Q1, Q3)	-	-	6.75 (5.48, 11.00)	-
Missing	-	-	0	
Cardiac fibrosis score, N (%)				0.053
F0	24 (42.9%)	1 (10.0%)	23 (50.0%)	
F1	20 (35.7%)	5 (50.0%)	15 (32.6%)	-
F2	8 (14.3%)	3 (30.0%)	5 (10.9%)	-
F3	4 (7.1%)	1 (10.0%)	3 (6.5%)	-
Pulmonary fibrosis score, N (%)				0.138
F0	22 (39.3%)	7 (70.0%)	15 (32.6%)	
F1	23 (41.1%)	3 (30.0%)	20 (43.5%)	
F2	9 (16.1%)	0 (0.0%)	9 (19.6%)	
F3	2 (3.6%)	0 (0.0%)	2 (4.3%)	

BSA = body surface area; F0 = fibrosis score 0; F1 = fibrosis score 1; F2 = fibrosis score 2; F3 = fibrosis score 3; IR = irradiated; NHP = nonhuman primate; PNC = pro-N-cadherin.

*P* values were obtained from Mann-Whitney *U* tests to compare continuous variables and from Fisher exact test for the categorical cardiac fibrosis score, across irradiation groups. Results are restricted to only NHPs with complete longitudinal serum PNC data.

### Cardiac fibrosis scores correlate with cardiac dysfunction of NHPs

**Figure 2:**
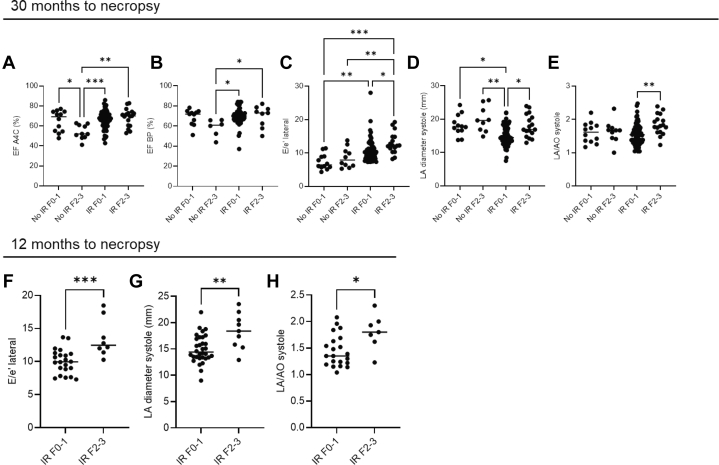
Echocardiogram Parameters by Subcohort (A) EF A4C = left ventricular ejection fraction apical 4-chamber, (B) EF BP = left ventricular ejection fraction biplane, (C) E/e’ lateral = ratio of mitral valve peak velocity to left ventricle peak tissue velocity E-Wave, (D) LA diameter systole = left atrium diameter systole – anteroposterior, (E) LA/AO systole = left atrium diameter to aortic root diameter – systole. A-E dots represent available echocardiogram examinations with corresponding serum samples drawn within 6 months of examination over 0 to 2 years prior to necropsy (30 months to necropsy) and are analyzed by nonparametric one-way Kruskal-Wallis test with Dunn’s post hoc tests. (F) E/e’ lateral = ratio of mitral valve peak velocity to left ventricle peak tissue velocity E-Wave, (G) LA diameter systole = left atrium diameter systole – anteroposterior, (H) LA/AO systole = left atrium diameter to aortic root diameter – systole. F-H dots represent individual NHPs and are analyzed by Mann-Whitney *U* test. The horizontal line represents the median value. Insufficient unirradiated NHP echocardiogram examinations within 12 months to necropsy for analysis. (∗*P* < 0.05; ∗∗*P* < 0.01; ∗∗∗*P* < 0.001).

Cardiac and pulmonary fibrosis were scored by board-certified pathologists using a 0 to 3 scale (F0 to F3), with F3 being the most severe (Figures 1A and 1B). To examine the cardiac function of NHPs that exhibited low (F0-1) vs high (F2-3) cardiac fibrosis scores, we analyzed echocardiogram parameters among 4 subcohorts (no IR F0-1, no IR F2-3, IR F0-1, and IR F2-3) (Figures 2A to 2H, Supplemental Tables 1 and 2). Notably, our results revealed distinct changes in ejection fractions within 30 months before necropsy among the no IR and IR cohorts. The values of the LV ejection fraction apical 2-chamber (EF A4C) were significantly decreased in no IR F2-3 NHPs compared to no IR F0-1 NHPs. However, no significant difference in EF A4C was observed between IR F0-1 and IR F2-3 NHPs (Figure 2A). A similar trend was also observed through the analysis of the values of LV ejection fraction biplane (Figure 2B). The ratio of mitral valve peak velocity to LV peak tissue velocity E-wave (E/e' lateral) was assessed within 30 months before necropsy and was significantly higher in IR cardiac F2-3 NHPs compared with any of the subcohorts (IR F2-3 vs IR F0-1, *P* < 0.05; IR F2-3 vs no IR F2-3, *P* < 0.01; IR F2-3 vs no IR F0-1, *P* < 0.001) (Figure 2C). The value of E/e' lateral assessed within 12 months before necropsy was also significantly higher in IR cardiac F2-3 NHPs compared to IR cardiac F0-1 NHPs (Figure 2F). In addition, the LA diameter at systole (LA diameter systole) was significantly increased in IR cardiac F2-3 NHPs compared with IR cardiac F0-1 NHPs (*P* < 0.05) within 30 and 12 months before necropsy, respectively (Figures 2D and 2G). Moreover, IR cardiac F2-3 NHPs exhibited a significant increase in the LA diameter to aortic root diameter–systole (LA/AO systole) compared to IR cardiac F0-1 NHPs within 30 and 12 months before necropsy, respectively (Figures 2E and 2H). The statistical significance of the above results was largely robust to adjustments for BSA and age at echocardiogram in the 30-month analysis and consistently robust in the 12-month analysis (Supplemental Tables 1 and 2). Together, these results reveal that while high cardiac fibrosis scores of no IR NHPs correlate with a decrease in EF, high cardiac fibrosis scores of IR NHPs correlate with an increase in diastolic LV filling pressure and diastolic dysfunction.

### Elevated serum PNC in IR NHP is associated with increased cardiac fibrosis

**Figure 3:**
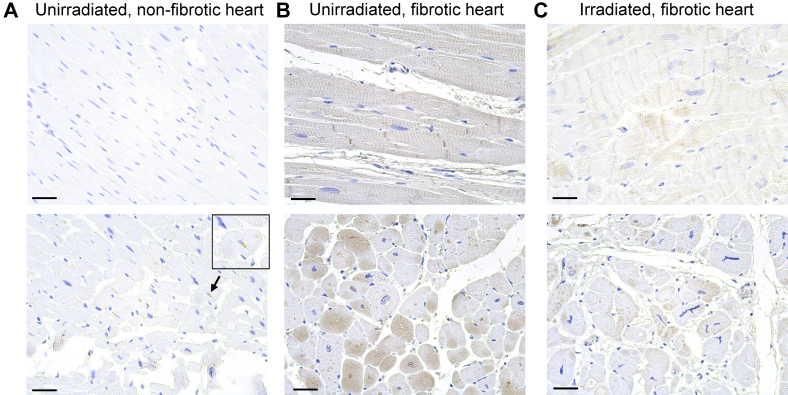
Aberrant Localization of Pro-N-Cadherin in Irradiated and Unirradiated Nonhuman Primate Fibrotic Cardiac Tissue Representative images of PNC staining (brown stain) of the hearts at necropsy from (A) unirradiated NHPs without cardiac fibrosis (F0), (B) unirradiated NHPs with cardiac fibrosis (F3), and (C) irradiated NHPs with cardiac fibrosis (F3). The top images are longitudinal sections, and the bottom images are transverse sections. Each image represents an individual NHP. In nonfibrotic cardiac tissues, perinuclear staining of PNC, consistent with normal N-cadherin processing, was observed (black arrow and inset zoomed image). In contrast, PNC IHC staining in fibrotic cardiac myocytes was present in a patchy cytoplasmic distribution, most intense at intercalated discs (B, top panel) and hypercontraction bands (C, top panel). Cardiac myocytes of affected animals showed other pathologies, including variation in myofiber diameter and karyomegaly, evident in panels B and C. Scale bars are 25 μm. IHC = immunohistochemistry; PNC = pro-N-cadherin; other abbreviation as in Figure 1.

To examine the expression of PNC in the heart of NHPs, cardiac tissues collected post-necropsy and independently scored for fibrosis were stained for PNC by IHC. Our results showed that the hearts of unirradiated NHPs without fibrosis (F0) exhibited perinuclear staining of PNC (Figure 3A). In contrast, PNC^+^ cells were substantially enriched in the hearts of unirradiated and IR NHPs that exhibited severe cardiac fibrosis (F3) (Figures 3B and 3C). PNC IHC staining in fibrotic cardiac myocytes was present in a patchy cytoplasmic distribution, most intense at intercalated discs (Figure 3B, top panel) and hypercontraction bands (Figure 3C, top panel). Cardiac myocytes of affected animals showed other pathologies, including variation in myofiber diameter, and karyomegaly, evident in Figures 3B and 3C. The patterns of aberrant localization of PNC are consistent with PNC localization observed in failing human hearts.^23^

**Figure 4:**
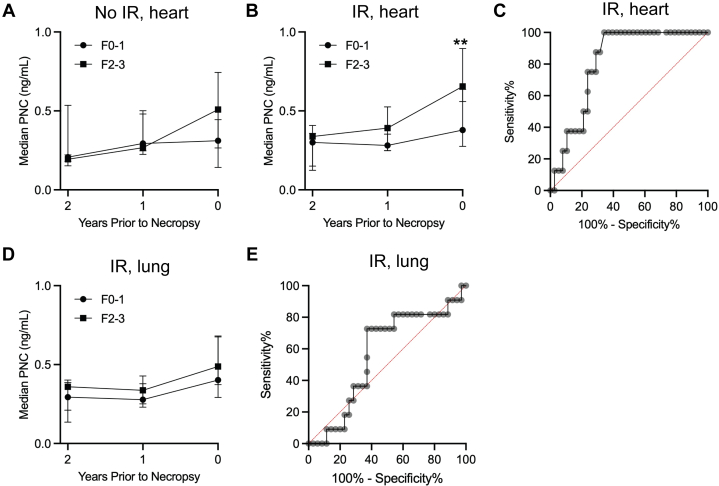
Elevated Serum Pro-N-Cadherin in Irradiated Nonhuman Primates With Cardiac Fibrosis (A) Longitudinal serum median PNC levels of unirradiated NHPs at 2 years, 1 year, and 0 year prior to necropsy stratified by cardiac fibrosis score at necropsy (F0-1 (N) = 6, F2-3 (N) = 4), error bars represent IQR. These differences were not significantly different by Mann-Whitney *U* test. (B) Longitudinal serum median PNC levels of irradiated NHPs at 2 years, 1 year, and 0 year prior to necropsy stratified by cardiac fibrosis score at necropsy (F0-1 (N) = 38, F2-3 (N) = 8), error bars represent IQR. Irradiated NHPs with cardiac fibrosis scores F2-3 have significantly higher serum PNC compared to irradiated NHPs with cardiac fibrosis scores F0-1 at necropsy by Mann-Whitney *U* test (*P* = 0.005). (C) ROC analysis of irradiated NHPs with cardiac fibrosis scores F0-1 relative to F2-3 at necropsy (IR F0-1, n = 38; IR F2-3, n = 8; AUC = 0.81; [95% CI: 0.69-0.93]; *P* = 0.006). (D) Longitudinal serum median PNC levels of irradiated NHPs at 2 years, 1 year, and 0 year prior to necropsy stratified by pulmonary fibrosis score at necropsy (F0-1 (N) = 35, F2-3 (N) = 11), error bars represent IQR. These differences were not significantly different by Mann-Whitney *U* test. (E) ROC analysis of irradiated NHPs with pulmonary fibrosis scores F0-1 relative to F2-3 at necropsy (IR F0-1, n = 35; IR F2-3, n = 11; AUC = 0.57; [95% CI: 0.38-0.76]; *P* = 0.511). (∗*P* < 0.05; ∗∗*P* < 0.01) ROC = receiver-operating characteristic; AUC = area under the curve; other abbreviations as in Figures 1 and 3.

Longitudinal serum samples 2, 1, and 0 years prior to necropsy were available from 10 unirradiated and 46 IR NHPs. Median serum PNC was higher in no IR cardiac F2-3 NHPs compared with no IR cardiac F0-1 NHPs at necropsy, although the difference was not statistically significant (Figure 4A). Serum PNC increased significantly (*P* = 0.005) in IR NHPs with more severe cardiac fibrosis, F2-3, at necropsy relative to IR NHPs with less severe cardiac fibrosis, F0-1 (Figure 4B). Receiver-operating characteristic analysis at necropsy of serum PNC demonstrated a significant discrepancy between IR NHPs with cardiac fibrosis scores F2-3 relative to IR NHPs with cardiac fibrosis scores F0-1 (area under the curve = 0.81, *P* = 0.006) (Figure 4C). No significant difference was observed between IR NHPs with pulmonary fibrosis scores F2-3 vs pulmonary fibrosis scores F0-1 at necropsy (Figures 4D and 4E). No severe pulmonary fibrosis (F2-3) was observed in unirradiated NHPs. Collectively, our findings support the hypothesis that serum PNC is a marker of cardiac fibrosis in IR NHPs.

### Serum PNC is associated with echocardiogram parameters of diastolic function

To determine the association between serum PNC and echocardiogram parameters, we examined echocardiogram measurements performed within 30 months of serum collection. The distribution of time of echocardiograms before necropsy was not significantly different between any of the subcohorts (Supplemental Figure 2). In unadjusted analyses within the IR subcohort (Table 2), higher log-transformed PNC showed significant associations with multiple markers of diastolic function, including E/e' lateral, the ratio of the peak early to late lateral mitral annular filling velocity (e’/a' lateral), LV early diastolic tissue velocity (e’ lateral), left atrial volume (LA Vol BP and LA Vol A2C), LVOT diam, and the ratio of peak early to late transmitral flow velocity in quartiles 3 to 4 (E/A Q3-Q4). Systolic and outflow measures were also affected, with significant associations across aortic valve measures (peak gradient, mean gradient, maximum velocity, mean velocity, and velocity time integral) and LV ejection fraction (EF A2C). Several associations demonstrated significant time interactions, indicating effects that became more pronounced approaching necropsy; for example, the effect of e' lateral at t_0_ was −0.11 (95% CI: −0.19 to −0.03), but attenuated by 0.07 (95% CI: 0.01-0.13) for each 1-year increase in time further from necropsy. After adjustment for BSA and age, findings were largely consistent, but DIFD, e’/a’ lateral, and EF A2C lateral no longer reached statistical significance. When this analysis was performed within the overall cohort (Supplemental Table 3), results were concordant, though E/e' lateral lost statistical significance and LVOT diam emerged as significant following covariate adjustment.

**Table 2:** Linear Mixed Model Results for the Association Between Log-Transformed PNC and Echocardiographic Measures, Within Irradiated NHPs Only

Echo Measure	Unadjusted Model^a^		Adjusted Model^b^		Sample Counts			
	Effect at Necropsy (Time 0)	Change in Effect per Year From Necropsy	Effect at Necropsy (Time 0)	Change in Effect per Year From Necropsy				
	Coefficient (95% CI)	Coefficient (95% CI)	Coefficient (95% CI)	Coefficient (95% CI)	N	n t0	n t1	n t2
AV PGmax	−0.14 (−0.22 to −0.07)^c^	0.09 (0.04-0.15)^d^	−0.12 (−0.20 to −0.04)^d^	0.09 (0.03-0.14)^d^	89	22	31	34
AV PGmean	−0.22 (−0.35 to −0.10)^d^	0.14 (0.04-0.25)^d^	−0.20 (−0.33 to −0.07)^d^	0.14 (0.04-0.24)^d^	89	22	31	34
AV Vmax	−0.78 (−1.39 to −0.17)^e^	0.33 (−0.11 to 0.77)	−0.69 (−1.32 to −0.05)^e^	0.36 (−0.08 to 0.80)	116	27	37	40
AV Vmean	−1.70 (−2.72 to −0.68)^d^	1.08 (0.31-1.86)^e^	−1.53 (−2.54 to −0.51)^d^	1.13 (0.37-1.88)^d^	89	22	31	34
AV VTI	−0.11 (−0.16 to −0.06)^c^	0.07 (0.03-0.11)^d^	−0.09 (−0.14 to −0.04)^d^	0.07 (0.03-0.11)^d^	89	22	31	34
DIFD	0.02 (0.00-0.05)^e^	−0.01 (−0.03 to 0.01)	0.02 (0.00-0.04)	−0.01 (−0.02 to 0.01)	88	22	31	33
e’/a’ lateral	−0.59 (−1.01 to −0.16)^d^	0.27 (−0.01 to 0.54)	−0.43 (−0.88 to 0.02)	0.24 (−0.04 to 0.52)	91	23	32	34
E/e’ lateral	0.08 (0.01-0.15)^e^	−0.05 (−0.10 to 0.00)	0.07 (0.00-0.13)^e^	−0.05 (−0.10 to 0.00)	90	23	32	33
EF A2C	−0.02 (−0.03 to 0.00)^e^	0.01 (−0.00 to 0.03)	−0.01 (−0.03 to 0.01)	0.01 (−0.00 to 0.02)	90	23	32	33
LA Vol A2C	0.17 (0.03-0.31)^e^	−0.13 (−0.23 to −0.03)^e^	0.16 (0.01-0.30)^e^	−0.12 (−0.22 to −0.02)^e^	90	22	32	34
LA Vol BP	0.19 (0.02-0.36)^e^	−0.14 (−0.25 to −0.03)^e^	0.17 (0.00-0.34)^e^	−0.13 (−0.24 to −0.02)^e^	88	39	20	32
LVOT diam	0.21 (0.01-0.41)^e^	−0.16 (−0.33 to −0.00)	0.22 (0.01-0.44)^e^	−0.18 (−0.35 to −0.02)^e^	89	22	32	33
e’ lateral	−0.11 (−0.19 to −0.03)^d^	0.07 (0.01-0.13)^e^	−0.10 (−0.18 to −0.02)^e^	0.08 (0.02-0.14)^e^	91	23	32	34
E/A (Q1-Q2)	−1.87 (−4.00 to 0.26)	0.42 (−1.06 to 1.89)	−1.68 (−3.90 to 0.54)	0.38 (−1.13 to 1.89)	51	13	21	15
E/A (Q3-Q4)	0.58 (0.21-0.95)^d^	−0.41 (−0.74 to −0.09)^e^	0.49 (0.08-0.89)^e^	−0.37 (−0.70 to −0.03)^e^	59	14	17	25

Within irradiated NHPs only (N = 46). Linear mixed models were fit with log-transformed PNC as the outcome, with echocardiogram measure, time, and their interaction as fixed effects, and a random intercept for subject to account for repeated measures. Coefficients with 95% CI represent the estimated change in log-transformed PNC per unit increase in echocardiogram measure at necropsy (Effect at Necropsy) and the change per year from necropsy in that association (Change in Effect per Year from Necropsy; echocardiogram × time interaction). Time coded as t0 = < 6 months from death, t1 = 6 to 18 months, t2 = 18 to 30 months. N denotes the total number of observations; n_t0_, n_t1_, and n_t2_ indicate the number of subjects with at least one observation at time points 0, 1, and 2, respectively.

Only parameters found significant in either unadjusted or adjusted models are included.

AV PGmax = aortic valve peak gradient; AV PGmean = aortic valve mean gradient; AV Vmean = aortic valve mean velocity; AV VTI = aortic valve velocity time integral; DIFD = left ventricle - major axis length diastolic difference - end diastole; e’ lateral = left ventricular peak early diastolic tissue velocity – lateral mitral annulus; e’/a’ lateral = ratio of the peak early to late lateral mitral annular filling velocity; E/e’ lateral = ratio of mitral valve peak velocity to left ventricle peak tissue velocity E-wave; EF A2C = left ventricular ejection fraction apical 2-chamber view; E/A (Q1-2) = ratio of the peak early to late transmitral flow velocity (quartiles 1 and 2); E/A (Q3-4) = ratio of the peak early to late transmitral flow velocity (quartiles 3 and 4); LA vol A2C = left atrial volume apical 2-chamber view – end systole; LA vol BP = left atrial volume biplane – end systole; LAA A2C = left atrial area apical 2-chamber view – end systole; LVOT diam = left ventricular outflow tract diameter; other abbreviations as in Table 1.

a Unadjusted models included echocardiogram measure, time, and their interaction term.

b Adjusted models additionally included body surface area and age at echocardiogram.

c *P* < 0.001. The Bonferroni-adjusted significance threshold was *P* < 0.001 (50 echocardiogram parameters tested; a subset of results is presented).

d *P* < 0.01.

e *P* < 0.05.

Together, these findings suggest that while serum PNC was not associated with all echocardiographic measures, its association with E/e′ lateral was comparable to the association between E/e’ lateral and high cardiac fibrosis score, with both relationships demonstrating statistical significance. Importantly, the association between serum PNC and E/e′ lateral remained after adjustment for age and BSA among IR NHPs (Table 2), highlighting the potential value of PNC as a serum biomarker of diastolic function.

### Associations of serum PNC with comorbidities of NHPs

Criteria for the diagnosis of comorbidities have previously been reported.^45^ In Table 3, in the IR subcohort, the high cardiac fibrosis group had a median PNC of 0.66 ng/mL at necropsy, compared to 0.38 ng/mL for the low cardiac fibrosis group, and these PNC distributions were significantly different (*P* = 0.006). After adjustment for BSA and age, no associations reached statistical significance, though cardiac fibrosis retained a strong association (*P* = 0.071). In the overall cohort (Supplemental Table 4), results were similar, with high cardiac fibrosis showing the largest median difference (0.28, *P* = 0.007), which remained statistically significant after adjustment (*P* = 0.023). In sum, these results indicate that, despite multi-organ injury caused by TBI, serum PNC is a marker of cardiac fibrosis of IR NHPs.

**Table 3:** Relationships Between PNC and Comorbidities at Necropsy, Within Irradiated NHPs Only

	Absent		Present		Median Diff.	*P* Value (Unadjusted)^a^	*P* Value (Adjusted for BSA)^a^	*P* Value (Adjusted for BSA and Age)^b^
	N	Median PNC (ng/mL) (Q1, Q3)	N	Median PNC (ng/mL) (Q1, Q3)				
Hypertension	31	0.41 (0.30, 0.63)	15	0.45 (0.24, 0.73)	0.04	0.888	0.923	0.914
Overweight	33	0.36 (0.28, 0.48)	13	0.64 (0.61, 0.85)	0.28	0.004^c^	0.122	0.122
Underweight	35	0.41 (0.29, 0.68)	11	0.43 (0.31, 0.49)	0.02	0.718	0.193	0.170
Tumor (carcinoma, sarcoma, both)	29	0.34 (0.25, 0.67)	17	0.49 (0.40, 0.78)	0.15	0.088	0.116	0.062
Gastrointestinal	29	0.48 (0.30, 0.68)	17	0.37 (0.25, 0.67)	−0.11	0.265	0.750	0.777
Heart murmur	14	0.37 (0.30, 0.58)	32	0.44 (0.29, 0.68)	0.07	0.858	0.918	0.762
Lung	32	0.40 (0.28, 0.71)	14	0.46 (0.36, 0.64)	0.06	0.793	0.566	0.655
Diabetes	32	0.40 (0.29, 0.67)	14	0.62 (0.31, 0.66)	0.21	0.431	0.357	0.457
Cataracts	15	0.49 (0.33, 0.79)	31	0.41 (0.24, 0.63)	−0.08	0.232	0.175	0.178
Hepatic cysts	35	0.40 (0.29, 0.63)	11	0.67 (0.38, 0.77)	0.27	0.181	0.416	0.511
Kidney	24	0.39 (0.25, 0.55)	22	0.56 (0.33, 0.76)	0.17	0.135	0.325	0.448
Arthritis	13	0.37 (0.29, 0.49)	33	0.45 (0.30, 0.68)	0.07	0.386	0.549	0.484
Dermatitis	26	0.41 (0.29, 0.62)	20	0.49 (0.32, 0.69)	0.09	0.565	0.891	0.760
Testicular atrophy	12	0.39 (0.31, 0.56)	34	0.44 (0.29, 0.68)	0.04	0.617	0.168	0.138
High cardiac fibrosis (2-3)	38	0.38 (0.29, 0.62)	8	0.66 (0.61, 0.87)	0.28	0.006^c^	0.059	0.071
High pulmonary fibrosis (2-3)	35	0.40 (0.29, 0.68)	11	0.49 (0.43, 0.66)	0.09	0.520	0.446	0.426

Within irradiated NHPs only (N = 46). Unadjusted *P* values are obtained from Mann-Whitney *U* tests comparing the distributions of PNC values at necropsy when the specified comorbidity is absent or present (ever).

∗*P* < 0.05.

CT = computed tomography; other abbreviations as in Table 1.

a Adjusted Model 1 reports *P* values from a proportional odds regression model adjusted for body surface area (BSA).

b Adjusted Model 2 additionally adjusts for age at necropsy using the same approach; this model was fit on a reduced sample as 6 NHPs had missing age at necropsy. Lung comorbidity was defined by abnormal CT density. Kidney comorbidity was defined by cyst, nephromegaly, abnormal CT density, or abnormal blood urea nitrogen (BUN) levels. Criteria for diagnosis of comorbidities have previously been reported.^45^

c *P* < 0.01. The Bonferroni-adjusted significance threshold was *P* < 0.003 (16 comorbidities tested).

## Discussion

Exposure to TBI is deleterious to all organ systems and often leads to comorbidities associated with or characterized by tissue fibrosis. Indeed, NHPs from this long-term survivor cohort carry comorbidities such as diabetes, hypertension, tumors, gastrointestinal disease, kidney disease, liver disease, and heart disease.^27^^,^^28^ It is noteworthy that despite multi-organ injury from TBI, PNC remains resilient to confounding comorbidities as a marker of cardiac fibrosis and diastolic echocardiogram parameters. However, the ideal timing for using serum PNC for RRHD detection or prediction remains to be established. Furthermore, while this study suggests organ specificity for diagnostic purposes in RRHD, our previous studies allude to PNC as a biomarker of HF from multiple etiologies.^22^, ^23^, ^24^

In the present study, IR NHPs with severe cardiac fibrosis exhibited significantly higher ejection fractions but also significantly elevated diastolic filling pressures compared to unirradiated animals with similar fibrosis scores. These findings support existing evidence that radiation exposure to the heart more commonly results in diastolic dysfunction, even when ejection fraction remains preserved. One possible explanation is that, although the severity of fibrosis appears comparable between IR and unirradiated animals with high cardiac fibrosis scores, differences in the distribution of fibrosis, collagen content, and collagen cross-linking may underlie the observed functional divergence.^46^, ^47^, ^48^, ^49^ On the contrary, echocardiogram data from unirradiated NHPs with high cardiac fibrosis scores were limited, and this observation could be due to a limited sample set.

Echocardiography is a noninvasive surrogate for measuring cardiac remodeling due to cardiac injury, stress, or disease. Low or high early E to late A ventricular filling velocities can indicate stiffness in the ventricular myocardium due to fibrotic remodeling. Elevated E/e’ values indicate increased LV filling pressures, which are also often due to fibrotic remodeling of the ventricle. Increased stiffness and prolonged exposure to elevated ventricle filling pressures during diastole lead to enlargement of the LA, increased LA volume, and left atrial area. Many of the echocardiogram parameters used in assessing diastolic dysfunction significantly correlate with serum PNC levels in these NHPs. Serum PNC levels could potentially be used as a surrogate measure of diastolic dysfunction. Several papers have examined the association between serological BNP/NT-proBNP and cardiotoxicity related to RT.^21^^,^^50^, ^51^, ^52^, ^53^, ^54^ For example, in a study using a cohort of long-term breast cancer survivors who received RT only, the changes in BNP levels were significantly higher in patients with increased cardiac and LV radiation doses.^51^ In another study including patients treated with thoracic RT, the percentage of patients with electrocardiogram changes among patients with elevated N-terminal pro-B-type natriuretic peptide (NT-proBNP) levels was significantly higher than that among patients without elevated NT-proBNP levels during the 6 to 12 months follow-up time. Studies evaluating traditional cardiac biomarkers in RRHD in Rhesus macaques are limited. One study demonstrated the utility of cardiac troponin I and NT-proBNP in identifying hypertrophic cardiomyopathy and other cardiac diseases in NHPs; however, these animals were not IR. Both cardiac troponin I and NT-proBNP had significant correlations with various echocardiogram parameters in this study.^55^ Although BNP/NT-proBNP serves the primary role of ruling out HF in emergency point-of-care settings and guiding treatment within the HF patient population,^19^ the unavoidable confounding variables and comorbidities found within the general population contribute to the lack of sensitivity or specificity of BNP/NT-proBNP as a biomarker for assessing the risk of HF.^56^, ^57^, ^58^ However, our prior work demonstrated that serological PNC levels are not affected by major confounders such as age, sex, and BMI,^22^ suggesting that PNC could offer advantages over BNP/NT-proBNP in predicting the risk of heart disease following radiation exposure.

### Study limitations

The inferences derived from this retrospective study are limited by the radiation exposure characteristics. Positive aspects of the exposure strategy include uniformity of the exposure field and well-controlled dose and dose rate. However, the uniformity of the exposure, while providing experimental rigor and reproducibility, would be unlikely in the case of a real-world exposure. The total radiation dose was lower than that for thoracic radiotherapy, and the dose was not fractionated. Relative to nuclear explosive devices, the total dose was higher than that experienced by survivors and consisted of photons only, not the mixed photon-proton-neutron spectrum likely in the case of a nuclear device. In addition, our results cannot exclude the possibility that fibrosis in organs other than the heart and lungs could potentially contribute to elevated serum PNC because not every organ was scored for fibrosis. While echocardiographic parameters provide a reasonable noninvasive assessment, invasive hemodynamic measurements remain the gold standard for evaluating diastolic filling pressures. Moreover, echocardiography was not performed in parallel with serum sample collection; therefore, correlations were conducted on echocardiogram measurements within 6 months of serum sample collection. The cohort consisted of only male Rhesus macaques. Serum samples from the unirradiated subcohorts were limited. As this work is exploratory in nature, the number of comparisons conducted relative to the available sample size limits the interpretability of individual *P* values, and many nominally significant associations do not survive multiple testing correction; nonetheless, the consistency and strength of the associations observed with cardiac fibrosis are notable and warrant further investigation in larger cohorts. Lastly, conventional echocardiogram threshold values defining diastolic dysfunction for Rhesus macaques have not been adequately defined in the literature.^59^

## Conclusions

Together, our findings demonstrate the significant utility of serum PNC for identifying cardiac fibrosis and significant correlations to echocardiogram parameters of diastolic dysfunction in RRHD in Rhesus macaques (Central Illustration). These findings suggest the promise of developing serum PNC as a biomarker of RRHD among cancer patients who receive thoracic radiotherapy and survivors of acute radiation exposure from nuclear accidents.

Perspectives **COMPETENCY IN MEDICAL KNOWLEDGE:** RRHD can be an undetected side effect of RT, especially in individuals undergoing treatment for cancers situated close to the chest, and has drawn interest in recent years. Yet, knowledge of its underlying mechanisms, detection, and effective treatment options remains incomplete. Identifying novel biomarkers for evaluating risk is vital for mitigating the effects of radiation exposure.

**TRANSLATIONAL OUTLOOK:** This study sets the stage for a continued effort to define qualifying biomarkers for Food and Drug Administration approval for evaluating RRHD. Future studies involving planned clinical trials with the inclusion of PNC and other biomarkers that utilize more clinically relevant radiation exposure protocols are critical for developing PNC as a biomarker of RRHD.

**Central Illustration:**
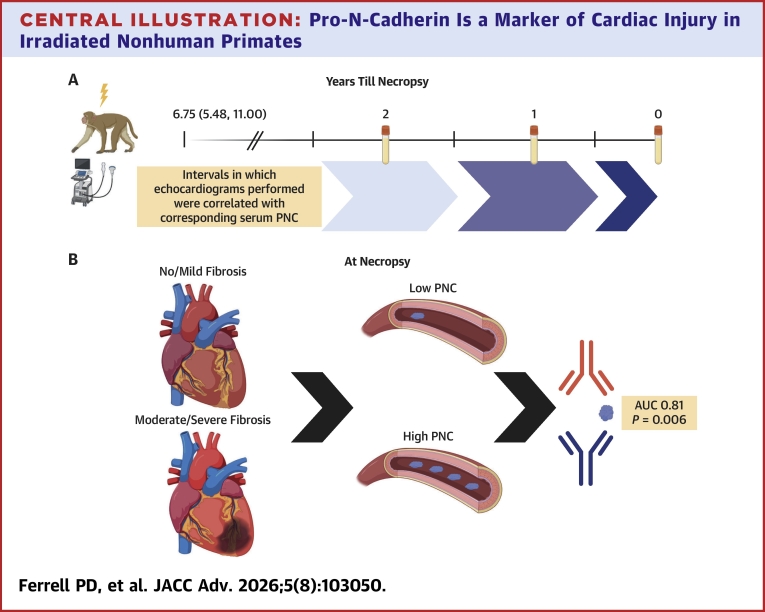
Pro-N-Cadherin Is a Marker of Cardiac Injury in Irradiated Nonhuman Primates The figure highlights a timeline for the retrospective analysis of available samples. (A) Illustration of median years from irradiation to necropsy, serum collection times prior to necropsy, and available echocardiograms correlated with serum pro-N-cadherin surrounding serum collection time points. (B) ROC analysis of pro-N-cadherin to detect moderate/severe cardiac fibrosis from post-necropsy evaluated cardiac tissues. The graphs were created with BioRender.com. Abbreviations as in Figures 1, 3, and 4.

## Funding support and author disclosures

The funding sources for this paper are 10.13039/100000002National Institutes of Health U01AI189426 (Dr Pizzo and Dr Lee), U01AI50578 (Dr Cline), U19AI67798 (Dr Cline), 5P30-CA014236-50 (Dr Niedzwiecki), DOD/CDMRP award W81XWH-15-1-0574 (Dr Cline and Dr Register), and Duke University School of Medicine Whitehead Scholar Award (Dr Lee). Mr Ferrell, Dr Oristian, and Dr Pizzo are inventors of unlicensed U.S. patent(s) on PNC held by Duke University. All other authors have reported that they have no relationships relevant to the contents of this paper to disclose.
