# Supplementary material for: Serum Pro-N-Cadherin Correlates With Cardiac Injury in the Radiation Late Effects Cohort of Nonhuman Primates
Source: JACC Adv. 2026 Jul 23;5(8):103050. doi: 10.1016/j.jacadv.2026.103050 (PMC13427542; doi:10.1016/j.jacadv.2026.103050)
Supplement: Supplemental Material [file mmc1.pdf]

Supplementary Materials

Serum pro-N-cadherin correlates with cardiac injury in the radiation late effects cohort of non-human primates

Supplementary Table 1. Echocardiogram summary between sub-cohorts, 30 months from necropsy……………..Page 2

Supplementary Table 2. Echocardiogram summary between sub-cohorts, 12 months from necropsy, within irradiated NHPs only...Page 5

Supplementary Table 3. Linear Mixed Model Results for the Association Between log-transformed PNC and Echocardiographic Measures..Page 7

Supplementary Table 4. Relationships between PNC and comorbidities at necropsy.........Page 9

Supplementary Figure 1. NHP plasma serial dilution fits non-linear regression

……………...Page 11

Supplementary Figure 2. Distribution of echocardiograms performed within 6 months of serum collection by sub-cohort…………………..Page 12

Supplementary Table 1. Echocardiogram summary between sub-cohorts, 30 months from necropsy

	NO IR F0-1	NO IR F2-3	IR F0-1	IR F2-3
**EF A4C**	69.19 (54.97, 74.26) * N=12	52.08 (49.31, 60.58) * €€€ ¥¥ N=10	67.31 (63.16, 71.70) €€€ N=72	70.06 (60.64, 72.87) ¥¥ N=17
**EF BP**	71.87 (64.08, 74.06) N=11	60.89 (54.24, 64.70) € ¥ N=6	69.92 (66.77, 73.64) € N=52	72.98 (62.43, 76.97) ¥ N=9
**DIFD**	10.74 (6.70, 15.09) Ω ‡ N=12	7.14 (4.60, 10.78) €€€ ¥¥¥ N=10	21.48 (14.50, 28.50) Ω €€€ N=71	24.45 (20.02, 28.17) ‡ ¥¥¥ N=17
**A’ LATERAL**	8.11 (6.87, 9.04) N=12	5.64 (4.27, 7.59) N=10	7.19 (5.69, 9.02) N=73	6.81 (5.75, 8.85) N=18
**A’ SEPTAL**	7.29 (6.20, 7.80) N=7	5.77 (5.11, 6.77) N=7	5.87 (4.90, 8.44) N=47	6.14 (6.07, 7.03) N=11
**E’ LATERAL**	12.04 (9.37, 12.88) N=12	11.62 (6.22, 13.47) N=10	10.17 (9.04, 11.99) N=73	9.47 (7.93, 10.34) N=18
**E/E’ LATERAL**	6.42 (5.77, 8.87) ΩΩ ‡‡‡ N=12	7.87 (6.26, 10.35) ¥¥ N=10	9.99 (8.68, 11.29) ΩΩ §§ N=73	12.25 (11.39, 14.83) ‡‡‡ ¥¥ §§ N=17
**E’/A’ LATERAL**	1.36 (1.23, 1.54) N=12	1.41 (1.18, 1.95) N=10	1.45 (1.15, 1.79) N=73	1.24 (1.01, 1.75) N=18
**E/A**	1.10 (0.97, 1.23) N=12	1.12 (0.98, 1.29) N=10	1.24 (1.09, 1.47) N=93	1.15 (0.93, 1.74) N=17

**MV E VEL**	0.79 (0.63, 0.82)<br>ΩΩ ‡‡<br>N=12	0.84 (0.74, 0.92)<br>€€€<br>N=10	1.01 (0.93, 1.17)<br>ΩΩ €€€<br>N=93	1.09 (0.91, 1.29)<br>‡‡<br>N=17
**LA VOL A2C**	3.37 (2.55, 4.65)<br>N=12	4.99 (3.17, 6.12)<br>€€€<br>N=10	2.26 (1.71, 2.97)<br>€€€<br>N=73	2.69 (1.73, 3.30)<br>N=17
**LA VOL BP**	4.05 (3.19, 5.13)<br>N=12	5.67 (3.93, 6.16)<br>€€€<br>N=10	3.11 (2.41, 3.69)<br>€€€<br>N=72	3.68 (3.13, 4.56)<br>N=16
**LAA A2C**	2.89 (2.51, 3.49)<br>Ω<br>N=12	3.54 (2.88, 3.92)<br>€€€ ¥<br>N=10	2.12 (1.70, 2.60)<br>Ω €€€<br>N=73	2.50 (1.71, 2.70)<br>¥<br>N=17
**LA DIAMETER SYSTOLE**	17.76 (16.62, 19.65)<br>ΩΩ<br>N=12	19.72 (17.40, 22.42)<br>€€€<br>N=10	14.36 (13.00, 17.12)<br>ΩΩ €€€ §§<br>N=97	17.14 (15.44, 19.35)<br>§§<br>N=22
**LA/AO SYSTOLE**	1.62 (1.36, 1.82)<br>N=12	1.67 (1.48, 1.73)<br>N=10	1.54 (1.35, 1.71)<br>§§<br>N=71	1.80 (1.62, 2.00)<br>§§<br>N=17

EF A4C = Left ventricular ejection fraction apical 2-chamber, EF BP = Left ventricular ejection fraction biplane, DIFD = Left ventricle - major axis length diastolic difference - end diastole, a' lateral = Left ventricle peak diastolic tissue velocity during atrial systole – lateral mitral annulus, a' septal = Left ventricle peak diastolic tissue velocity during atrial systole – septal mitral annulus, e' lateral = Left ventricular peak early diastolic tissue velocity – lateral mitral annulus, E/e' lateral = ratio of mitral valve peak velocity to left ventricle peak tissue velocity E-Wave, e'/a' lateral = ratio of the peak early to late lateral mitral annular filling velocity, E/A = ratio of the peak early to late transmitral flow velocity, MV E vel = Mitral valve E wave peak velocity, LA vol A2C = Left atrial volume apical 2-chamber – end systole, LA vol BP = Left atrial volume biplane – end systole, LAA A2C = Left atrial area apical 2-chamber – end systole, LA diameter systole = Left atrium diameter systole – antero-posterior, LA/AO systole = Left atrium diameter to aortic root diameter – systole. Overall cohort number of NHPs = 56, counts (N) represent total echocardiogram examinations for each measure with corresponding serum samples within 6 months of examination over 0-2 years prior to necropsy. Median (Q1, Q3) values are presented. P-values were calculated from one-way Kruskal-Wallis tests across strata for each measure,

with Dunn's post-hoc tests (* $p < 0.05$; ** $p < 0.01$; *** $p < 0.001$), (€ $p < 0.05$; €€ $p < 0.01$; €€€ $p < 0.001$), (¥ $p < 0.05$; ¥¥ $p < 0.01$; ¥¥¥ $p < 0.001$), (Ω $p < 0.05$; Ω Ω $p < 0.01$; Ω Ω Ω $p < 0.001$), (‡ $p < 0.05$; ‡‡ $p < 0.01$; ‡‡‡ $p < 0.001$), (§ $p < 0.05$; §§ $p < 0.01$; §§§ $p < 0.001$). Matching symbols indicate significance between the groups. Underlined symbols indicate pairwise comparisons that retained statistical significance ($p < 0.05$) after proportional odds regression adjusted for BSA and age at echocardiogram. The Bonferroni-adjusted significance threshold was $p < 0.001$ (50 echocardiogram parameters tested; a subset of results is presented).

Supplementary Table 2. Echocardiogram summary between sub-cohorts, 12 months from necropsy, within irradiated NHPs only.

	IR F0-1	IR F2-3	P-VALUE (UNADJUSTED)[1]	P-VALUE (ADJUSTED)[2]
**EF A4C**	68.21 (64.06, 74.52) N=22	69.47 (58.60, 72.66) N=7	0.746	0.622
**EF BP**	70.05 (67.08, 75.06) N=21	72.26 (60.08, 73.58) N=7	0.466	0.813
**DIFD**	22.65 (16.15, 30.61) N=22	25.70 (20.57, 30.08) N=7	0.636	0.828
**A' LATERAL**	7.72 (6.57, 9.47) N=22	8.42 (5.96, 10.22) N=8	0.784	0.596
**A' SEPTAL**	7.92 (5.13, 9.01) N=20	6.67 (6.41, 7.03) N=3	0.635	0.254
**E' LATERAL**	10.71 (9.51, 11.78) N=22	9.70 (8.46, 10.26) N=8	0.079	0.387
**E/E' LATERAL**	9.96 (8.90, 11.12) N=22	12.47 (11.85, 14.56) N=8	<0.001***	0.003**
**E'/A' LATERAL**	1.37 (1.18, 1.63) N=22	1.09 (0.76, 1.48) N=8	0.217	0.942
**E/A**	1.13 (0.98, 1.25) N=31	1.00 (0.90, 1.95) N=8	0.983	0.49
**MV E VEL**	0.99 (0.91, 1.16) N=31	1.19 (0.99, 1.40) N=8	0.330	0.152
**LA VOL A2C**	2.30 (2.19, 2.89) N=22	2.49 (1.91, 3.61) N=7	>0.999	0.861

**LA VOL BP**	3.00 (2.69, 3.47)<br>N=21	3.68 (2.66, 4.34)<br>N=6	0.263	0.149
**LAA A2C**	2.20 (2.06, 2.57)<br>N=22	2.30 (1.84, 2.92)<br>N=7	>0.999	0.710
**LA DIAMETER SYSTOLE**	14.42 (13.40, 17.00)<br>N=32	18.40 (15.85, 20.50)<br>N=9	0.027*	0.008**
**LA/AO SYSTOLE**	1.35 (1.22, 1.63)<br>N=21	1.80 (1.68, 1.97)<br>N=7	0.030*	0.016*

EF A4C = Left ventricular ejection fraction apical 2-chamber, EF BP = Left ventricular ejection fraction biplane, DIFD = Left ventricle - major axis length diastolic difference - end diastole, a' lateral = Left ventricle peak diastolic tissue velocity during atrial systole – lateral mitral annulus, a' septal = Left ventricle peak diastolic tissue velocity during atrial systole – septal mitral annulus, e' lateral = Left ventricular peak early diastolic tissue velocity – lateral mitral annulus, E/e' lateral = ratio of mitral valve peak velocity to left ventricle peak tissue velocity E-Wave, e'/a' lateral = ratio of the peak early to late lateral mitral annular filling velocity, E/A = ratio of the peak early to late transmitral flow velocity, MV E vel = Mitral valve E wave peak velocity, LA vol A2C = Left atrial volume apical 2-chamber – end systole, LA vol BP = Left atrial volume biplane – end systole, LAA A2C = Left atrial area apical 2-chamber – end systole, LA diameter systole = Left atrium diameter systole – antero-posterior, LA/AO systole = Left atrium diameter to aortic root diameter – systole. Irradiated cohort number of NHPs = 46, counts (N) represent total echocardiogram examinations with corresponding serum samples for each measure within 6 months of examination over 12 months prior to necropsy. Median (Q1, Q3) values are presented. Unadjusted p-values[1] were obtained from Mann-Whitney U tests across strata for each measure. Adjusted p-values[2] were obtained from proportional odds regression models adjusting for BSA and age at echocardiogram. (* $p < 0.05$; ** $p < 0.01$; *** $p < 0.001$). The Bonferroni-adjusted significance threshold was $p < 0.001$ (50 echocardiogram parameters tested; a subset of results is presented).

**Supplementary Table 3. Linear Mixed Model Results for the Association Between log-transformed PNC and Echocardiographic Measures.**								
	**Unadjusted Model[1]**		**Adjusted Model[2]**		**Sample Counts**			
	**Effect at Necropsy (time 0)**	**Change in Effect per Year from Necropsy**	**Effect at Necropsy (time 0)**	**Change in Effect per Year from Necropsy**				
**Echo Measure**	**Coefficient (95% CI)**	**Coefficient (95% CI)**	**Coefficient (95% CI)**	**Coefficient (95% CI)**	**N**	**n t0**	**n t1**	**n t2**
AV PGmax	-0.13 (-0.20, -0.06) ***	0.07 (0.03, 0.12) **	-0.11 (-0.18, -0.04) **	0.07 (0.02, 0.12) **	108	24	38	43
AV PGmean	-0.20 (-0.32, -0.09) **	0.11 (0.02, 0.20) *	-0.17 (-0.29, -0.06) **	0.11 (0.02, 0.20) *	108	24	38	43
AV Vmax	-0.83 (-1.39, -0.26) **	0.31 (-0.08, 0.70)	-0.70 (-1.28, -0.12) *	0.29 (-0.10, 0.68)	135	29	44	49
AV Vmean	-1.56 (-2.49, -0.64) **	0.81 (0.15, 1.47) *	-1.31 (-2.24, -0.38) **	0.82 (0.17, 1.48) *	108	24	38	43
AV VTI	-0.10 (-0.15, -0.06) ***	0.06 (0.03, 0.09) **	-0.09 (-0.14, -0.04) **	0.06 (0.03, 0.09) **	108	24	38	43
DIFD	0.02 (-0.00, 0.04)	-0.01 (-0.02, 0.01)	0.01 (-0.01, 0.03)	0.00 (-0.02, 0.01)	110	25	39	43
e'/a' lateral	-0.49 (-0.88, -0.11) *	0.21 (-0.03, 0.45)	-0.35 (-0.75, 0.05)	0.17 (-0.08, 0.41)	109	25	39	42
E/e' lateral	0.06 (0.00, 0.12) *	-0.03 (-0.08, 0.01)	0.05 (-0.00, 0.11)	-0.03 (-0.07, 0.01)	109	25	39	42
EF A2C	-0.01 (-0.03, 0.01)	0.01 (-0.01, 0.02)	-0.01 (-0.03, -0.00) *	0.01 (-0.00, 0.02)	109	24	39	43
LA Vol A2C	0.12 (0.01, 0.23) *	-0.10 (-0.17, -0.02) *	0.12 (0.01, 0.23) *	-0.09 (-0.17, -0.01) *	107	22	39	43
LA Vol BP	0.14 (0.01, 0.26) *	-0.10 (-0.18, -0.01) *	0.13 (0.00, 0.25) *	-0.09 (-0.18, -0.01) *	109	24	39	43
LVOT diam	0.14 (-0.02, 0.30)	-0.11 (-0.22, 0.00)	0.17 (0.01, 0.34) *	-0.13 (-0.24, -0.01) *	110	25	39	43
e' lateral	-0.10 (-0.17, -0.03) **	0.05 (-0.00, 0.10)	-0.08 (-0.15, -0.01) *	0.04 (-0.01, 0.09)	63	14	27	19
E/A (Q1-Q2)	-1.63 (-3.46, 0.20)	0.23 (-1.06, 1.52)	-1.41 (-3.28, 0.45)	0.23 (-1.07, 1.53)	66	15	18	30
E/A (Q3-Q4)	0.55 (0.20, 0.90) **	-0.35 (-0.60, -0.11) **	0.52 (0.15, 0.90) **	-0.35 (-0.60, -0.10) **	108	24	39	42
Overall cohort N = 56. Linear mixed models were fit with log-transformed PNC as the outcome, with echocardiogram measure, time, and their interaction as fixed effects, and a random intercept for subject to account for repeated measures. Coefficients with 95% CI represent the estimated change in log-transformed PNC per unit increase in echocardiogram measure at necropsy (Effect at Necropsy) and the change per year from necropsy in that association (Change in Effect per Year from Necropsy; echocardiogram × time interaction). Time coded as t0 = < 6 months from death, t1 = 6–18 months, t2 = 18-								

30 months. N denotes the total number of observations; n_{t0}, n_{t1}, and n_{t2} indicate the number of subjects with at least one observation at time points 0, 1, and 2, respectively.
[1]Unadjusted models included echocardiogram measure, time, and their interaction term.
[2]Adjusted models additionally included body surface area and age at echocardiogram.
Only parameters found significant in either unadjusted or adjusted models are included. AV PGmax = Aortic valve peak gradient, AV PGmean = Aortic valve mean gradient, AV Vmean = Aortic valve mean velocity, AV VTI = Aortic valve velocity time integral, DIFD = Left ventricle - major axis length diastolic difference - end diastole, e' lateral = Left ventricular peak early diastolic tissue velocity – lateral mitral annulus, e'/a' lateral = ratio of the peak early to late lateral mitral annular filling velocity, E/e' lateral = Ratio of mitral valve peak velocity to left ventricle peak tissue velocity E-Wave, EF A2C = Left ventricular ejection fraction apical 2-chamber view, E/A (Q1-2) = ratio of the peak early to late transmitral flow velocity (Quartiles 1 and 2), E/A (Q3-4) = ratio of the peak early to late transmitral flow velocity (Quartiles 3 and 4), LA vol A2C = Left atrial volume apical 2-chamber view – end systole, LA vol BP = Left atrial volume biplane – end systole, LAA A2C = Left atrial area apical 2-chamber view – end systole, LVOT diam = Left ventricular outflow tract diameter.
* $p < 0.05$; ** $p < 0.01$; *** $p < 0.001$ and significant after Bonferroni correction (50 echocardiogram parameters tested)

Supplementary Table 4. Relationships between PNC and comorbidities at necropsy.								
Comorbidity	Absent		Present		Median Diff.	p-value (unadjusted)[1]	p-value (adjusted for BSA)[1]	p-value (adjusted for BSA and age)[2]
	N	Median PNC (ng/mL) (Q1, Q3)	N	Median PNC (ng/mL) (Q1, Q3)				
Hypertension	39	0.40 (0.29, 0.62)	17	0.45 (0.23, 0.77)	0.04	0.803	0.906	0.809
Overweight	37	0.37 (0.28, 0.48)	19	0.62 (0.39, 0.72)	0.25	0.072	0.176	0.186
Underweight	43	0.40 (0.29, 0.67)	13	0.43 (0.30, 0.48)	0.02	0.801	0.790	0.614
Tumor (carcinoma, sarcoma, both)	38	0.35 (0.24, 0.66)	18	0.49 (0.39, 0.74)	0.13	0.120	0.091	0.032*
Gastrointestinal	38	0.44 (0.29, 0.66)	18	0.39 (0.26, 0.64)	-0.05	0.488	0.789	0.895
Heart murmur	31	0.39 (0.29, 0.65)	25	0.41 (0.23, 0.68)	0.05	0.961	0.651	0.964
Lung	38	0.40 (0.26, 0.66)	18	0.44 (0.31, 0.64)	0.03	1.000	0.963	0.945
Diabetes	40	0.40 (0.29, 0.67)	16	0.57 (0.22, 0.64)	0.17	0.790	0.785	0.942
Cataracts	21	0.36 (0.30, 0.68)	35	0.41 (0.24, 0.63)	0.05	0.710	0.700	0.704
Hepatic cysts	44	0.39 (0.28, 0.63)	12	0.58 (0.39, 0.72)	0.19	0.122	0.132	0.240
Kidney	32	0.39 (0.24, 0.55)	24	0.47 (0.32, 0.71)	0.08	0.145	0.143	0.330
Arthritis	15	0.37 (0.27, 0.49)	41	0.43 (0.30, 0.67)	0.06	0.350	0.682	0.900
Dermatitis	32	0.39 (0.25, 0.52)	24	0.51 (0.32, 0.69)	0.12	0.289	0.367	0.638
Testicular atrophy	21	0.38 (0.24, 0.48)	35	0.45 (0.29, 0.69)	0.06	0.163	0.057	0.017*

Irradiated	10	0.37 (0.23, 0.47)	46	0.42 (0.29, 0.67)	0.05	0.252	0.053	0.037*
High cardiac fibrosis (2-3)	44	0.38 (0.25, 0.52)	12	0.65 (0.52, 0.79)	0.28	0.007**	0.018*	0.023**
High pulmonary fibrosis (2-3)	45	0.39 (0.28, 0.67)	11	0.49 (0.43, 0.66)	0.10	0.343	0.256	0.227

Overall cohort N = 56. Unadjusted P-values are obtained from Mann-Whitney U tests comparing the distributions of PNC values at necropsy when the specified comorbidity is absent or present (ever). Adjusted Model 1 reports p-values from a proportional odds regression model adjusted for body surface area (BSA). Adjusted Model 2 additionally adjusts for age at necropsy using the same approach; this model was fit on a reduced sample as 6 NHPs had missing age at necropsy. Lung comorbidity was defined by abnormal CT density. Kidney comorbidity was defined by cyst, nephromegaly, abnormal CT density, or abnormal blood urea nitrogen (BUN) levels. Criteria for diagnosis of comorbidities has previously been reported[47]. * $p < 0.05$; ** $p < 0.01$. The Bonferroni-adjusted significance threshold was $p < 0.003$ (17 comorbidities tested).

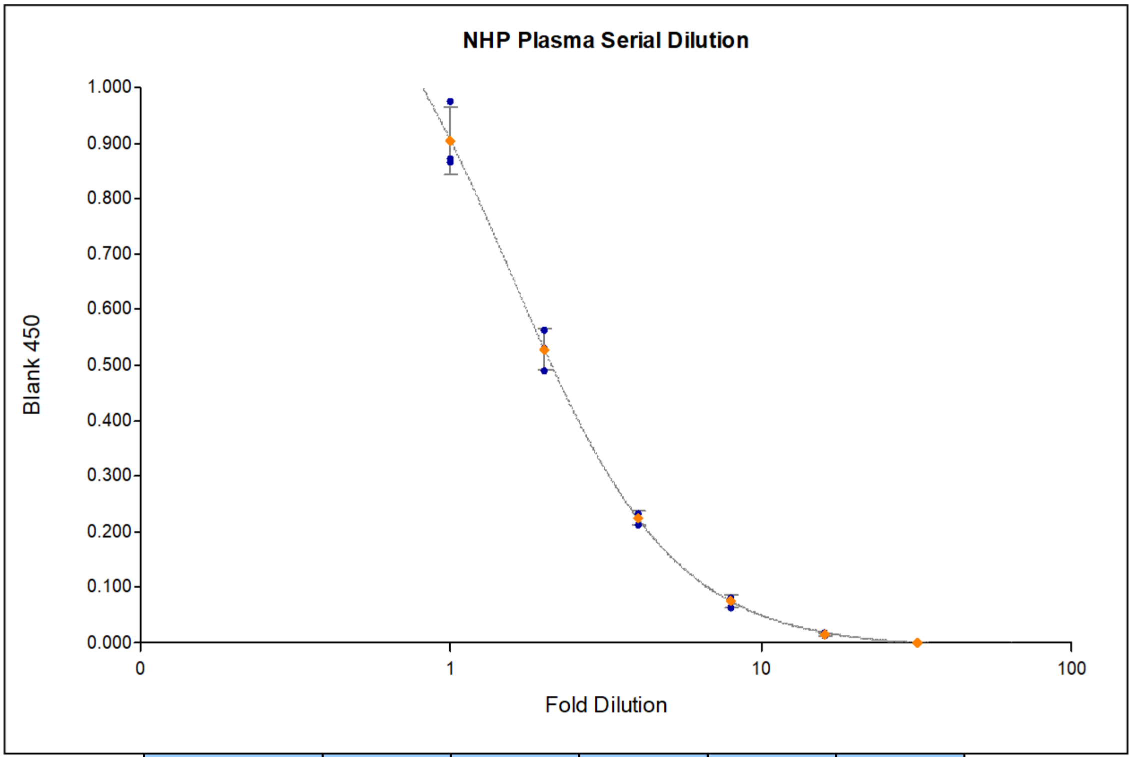

Curve Formula	A	B	C	D	R2
Y = (A-D)/(1+(X/C)^B) + D	1.34	1.67	1.57	-0.00894	1

Supplementary Figure 1. NHP plasma serial dilution fits non-linear regression. The pooled NHP plasma sample was serially diluted in triplicate and quantified by PNC ELISA using previously established methods (r^2 = 1.00). Dilutions started with neat (1-fold), followed by 2-fold, 4-fold, 8-fold, 16-fold, and 32-fold.

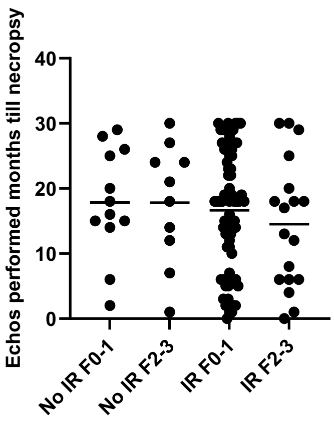

Supplementary Figure 2. Distribution of echocardiograms performed within 6 months of serum collection by sub-cohort. The distribution of time of echocardiograms prior to necropsy was not significantly different between any of the sub-cohorts. (No IR F0-1 vs. No IR F2-3, $p>0.999$; No IR F0-1 vs. IR F0-1, $p=0.976$; No IR F0-1 vs. IR F2-3, $p=0.768$; No IR F2-3 vs. IR F0-1, $p=0.983$; No IR F2-3 vs. IR F2-3, $p=0.802$; IR F0-1 vs. IR F2-3, $p=0.815$)
